# Effect of electroacupuncture on opioid consumption in patients with chronic musculoskeletal pain: protocol of a randomised controlled trial

**DOI:** 10.1186/1745-6215-13-169

**Published:** 2012-09-17

**Authors:** Charlie CL Xue, Robert D Helme, Stephen Gibson, Malcolm Hogg, Carolyn Arnold, Andrew A Somogyi, Cliff Da Costa, Yanyi Wang, Shao-chen Lu, Zhen Zheng

**Affiliations:** 1 Traditional and Complementary Medicine Research Program, Health Innovations Research Institute (HIRi), School of Health Sciences, RMIT University, Bundoora, VIC, Australia; 2 Department of Medicine, Royal Melbourne Hospital, Parkville, VIC, Australia; 3 National Ageing Research Institute, Parkville, VIC, Australia; 4 Pain Services, Royal Melbourne Hospital, Parkville, VIC, Australia; 5 Caulfield Pain Management and Research Centre, Caulfield Hospital, Caulfield, VIC, Australia; 6 Discipline of Pharmacology, School of Medical Sciences, University of Adelaide, Adelaide, SA, Australia; 7 School of Mathematical and Geospatial Sciences, RMIT University, Bundoora, VIC, Australia

**Keywords:** Acupuncture, Chronic pain, Electroacupuncture, Opioid medication, Pain education, Randomised controlled trial

## Abstract

**Background:**

Chronic musculoskeletal pain is common and has been increasingly managed by opioid medications, of which the long-term efficacy is unknown. Furthermore, there is evidence that long-term use of opioids is associated with reduced pain control, declining physical function and quality of life, and could hinder the goals of integrated pain management. Electroacupuncture (EA) has been shown to be effective in reducing postoperative opioid consumption. Limited evidence suggests that acupuncture could assist patients with chronic pain to reduce their requirements for opioids.

The proposed research aims to assess if EA is an effective adjunct therapy to standard pain and medication management in reducing opioids use by patients with chronic musculoskeletal pain.

**Methods:**

In this multicentre, randomised, sham-acupuncture controlled, three-arm clinical trial, 316 patients regularly taking opioids for pain control and meeting the defined selection criteria will be recruited from pain management centres and clinics of primary care providers in Victoria, Australia. After a four-week run-in period, the participants are randomly assigned to one of three treatment groups to receive EA, sham EA or no-EA with a ratio of 2:1:1. All participants receive routine pain medication management delivered and supervised by the trial medical doctors. Twelve sessions of semi-structured EA or sham EA treatment are delivered over 10 weeks. Upon completion of the acupuncture treatment period, there is a 12-week follow-up. In total, participants are involved in the trial for 26 weeks. Outcome measures of opioid and non-opioid medication consumption, pain scores and opioid-related adverse events are documented throughout the study. Quality of life, depression, function, and attitude to pain medications are also assessed.

**Discussion:**

This randomised controlled trial will determine whether EA is of significant clinical value in assisting the management of debilitating chronic pain by reducing opioids consumption and their associated adverse events, as well as improving the quality of life for those with chronic pain. Such an outcome will provide the rationale for including EA into multidisciplinary programmes for effective management of chronic musculoskeletal pain.

**Trial registration:**

Australian New Zealand Clinical Trial Registry (ACTRN12609000676213)

http://www.anzctr.org.au/trial_view.aspx?ID=308008

## Background

### Opioid medications for chronic pain

One in five Australians suffers from chronic pain, with the majority having chronic musculoskeletal pain (CMP) [1]. Chronic pain is the third most costly health condition and costs the Australian government $4.7 billion dollars each year [2].

Due to the difficulty in managing CMP, morphine and other strong opioids have been increasingly used. Opioid medication (OM) prescriptions for CMP doubled from 8% to 16% in the United States of America between 1980 and 2000 [3]. In Australia, 13% of chronic pain patients were on some type of opioids in a 1999 survey [4]. From 1999 to 2005, the total OM consumption in Australia had increased by more than eight times for oxycodone (to 38.9 mg per capita), 40 times for hydromorphone and five times for fentanyl [5].

The effectiveness of OM for CMP is debatable [6]. Three recent systematic reviews concluded that OM reduced pain by 30% in the short term. However, its long-term efficacy is unknown [7-9]. A follow-up study of patients who were still on OM 10 years after completion of a multidisciplinary pain management programme reported that those patients had poorer quality of life, were more depressed, and adopted passive strategies for pain management, indicating that opioids were ineffective for this group [10]. Furthermore, opioids are associated with a high rate of adverse events (AEs), such as drowsiness, nausea, vomiting, constipation, dry mouth, dose escalation and/or tolerance, hyperalgesia and dependence when used for treating chronic pain [11].

Part of integrated pain management is to assist patients in reducing OM use through pain medication management (PMM), including education about pain and adjustment of pain medication, and is usually given by clinicians and nurses. However, there are very few other options. There is an urgent need to identify a therapy that could effectively assist the reduction of OM.

### Electroacupuncture

Acupuncture, one of several non-pharmacological therapies for managing pain, has been found to stimulate the releases of endogenous opioid peptides in animals and humans [12-14]. A recent human study using positron emission tomography found that patients with fibromyalgia displayed reduced binding potential of opioids in comparison to pain-free controls [15]. In addition, nine sessions of acupuncture increased opioid binding potential in certain brain regions of a similar group of patients significantly more than sham acupuncture [16]. Combined with the increased release of endogenous opioid peptides upon acupuncture, this increased binding effect might in turn enhance the efficiency of OM and decrease the amount required for functional improvement and pain relief.

When used in postoperative pain, electroacupuncture (EA) - acupuncture combined with electrical stimulation - reduced the use of postoperative morphine by more than 60% and was 30% better than sham procedures. It also reduced the severity and incidence of OM-related side effects [17,18]. Our pilot study in 35 patients with chronic pain also showed that EA reduced OM consumption by 39% in comparison with a 25% reduction in the sham EA group, with 90% patient satisfaction [19].

### Aims

In this multicentre, randomised, sham-acupuncture controlled, three-arm clinical trial, we will evaluate the effect of EA as an adjunct therapy to PMM on OM consumption by patients with CMP. The primary hypothesis is that EA reduces OM consumption significantly more than sham EA. The secondary hypotheses are that:

1. the proportion of participants who experience 50% or more OM reduction is significantly higher in the EA group than that in the sham EA group;

2. EA reduces OM-related AEs significantly more than sham EA;

3. EA combined with PMM is significantly better than PMM alone in reducing OM usage and AEs;

4. EA reduces the intensity of pain and improves function significantly better than sham EA;

5. participants having a strong self-reliant attitude to pain consume significantly less OM than those relying on medications for pain relief.

## Methods

The trial is being conducted between July 2009 and December 2012, and has been approved by the Human Research Ethics Committees of Melbourne Health (2009.033), Alfred Health (80.09) and the RMIT University (06/09) to be conducted at the Pain Services of the Royal Melbourne Hospital, the Caulfield Pain Management and Research Centre of the Caulfield Hospital, the Sunshine Hospital, and various sites in Melbourne and Geelong. It has been registered with the Australian New Zealand Clinical Trial Registry (ACTRN12609000676213).

**Figure 1:**
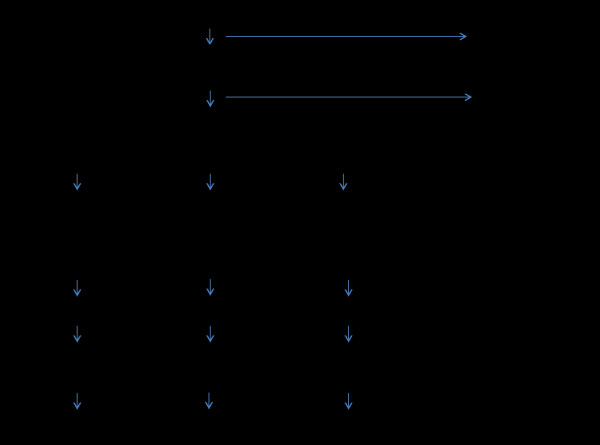
A flow chart of the trial process.

The design of the trial follows the CONSORT statement [20] and the Initiative on Methods, Measurements, and Pain Assessment in Clinical Trial for chronic pain [21]. The procedure of the trial is presented in Figure 1.

### Power and sample size calculation

Using sample means from baseline and the end of the treatment phase of the OM consumption data from our pilot study [19], we estimate that a total sample size of 316 will provide 80% power using a 1% level of significance to detect an effect size estimate of 0.48 between the EA and sham EA groups. Because intention-to-treat analysis will be used, no additional participants are required. We plan to recruit 316 participants in total. A dropout rate of 20% is considered to be within the normal range.

### Participant recruitment

Patients attending three major pain management centres are notified of the trial by leaflets displayed at the centres or referred by the staff at the pain clinics. Information about the trial is also disseminated through health professionals’ networks, including Chinese medicine, chiropractic, general practice, massage therapy, naturopathy, osteopathy, pharmacy and physiotherapy and through networks for patients with chronic pain, such as Chronic Pain Australia. Interested patients complete a form to express their intention to participate. A researcher from the trial administration centre (RMIT University) then conducts an initial screening, which is followed by a face-to-face interview with a researcher and assessment by a medical doctor (MD) at one of the hospital sites prior to randomisation.

#### *Inclusion criteria*

Participants meeting the following criteria are included:

1. age between 18 and 85 years at entry

2. confident in conversational and reading English

3. suffering from CMP, regardless of the locations of pain

4. have taken OM regularly for more than two months without dose limitation.

#### *Exclusion criteria*

Participants having one or more of the following criteria are excluded:

1. active abuse of OM as judged by an MD

2. severely depressed with suicidal tendency as judged by MDs

3. unstable heart condition, pregnancy or intent to become pregnant, breastfeeding women, epilepsy, brain tumour, current cancer, haemophilia or wearing cardiac pacemakers

4. no general practitioner available for liaison

5. acupuncture treatment in the last 12 months

6. unwilling to reduce OM.

Prior to the eligibility assessment, all participants are provided with adequate information about the study, written in plain English, and all questions are answered to their level of satisfaction before informed consent is signed.

### Randomisation and blinding procedures

After a four-week run-in period, participants who meet all selection criteria are randomly allocated into one of the three groups, namely EA, sham EA or no-EA with a 2:1:1 ratio. An automated telephone randomisation service provided by the National Health and Medical Research Council Clinical Trial Centre at the University of Sydney is used. Before the commencement of the first acupuncture session, the treating acupuncturist dials a toll-free number and enters the identification codes of the trial, the acupuncturist and the site, and the baseline OM weekly dose (morphine equivalent). The participant is then randomly allocated to one of the three groups according to a computer-generated randomisation sequence. The acupuncturist records the allocation in a secure and separate file. The computer-generated randomisation uses stratified permuted blocks to ensure that an equal ratio of participants in EA and sham EA groups in each centre is assigned to each acupuncturist; and to ensure that an equal number of participants consuming high or low dose of OM is assigned to each group. The cut-off OM dose for high or low consumption is 560 mg (morphine equivalents) per week, decided upon by pain specialists on the team based on our pilot study and other published studies reporting average OM consumption by patients with chronic pain [22,23]. Three quarters of the participants are in either the EA or sham EA group and are blinded; one quarter of the participants are in the no-EA group and are be blinded. The success of blinding of the two acupuncture groups is assessed. Details are presented in Assessment of efficacy below.

The acupuncturists and MDs are not involved in outcome assessments. Blinded assessors are required to conduct outcome assessments, collect Medication and Pain Diaries, and check any missing data. The trial data are entered by research assistants and analysed by a statistician. Except for the acupuncturists, all of the above will be are blinded to participant allocation. Professional statistical assistance is also sought to double check the data analysis.

### Trial procedure

As shown in Figure 1, during the initial visit participants provide personal and demographic details and pain history. A four-week run-in period (first to fourth week) follows. During this time, the general practitioners of the participants are notified. Their assistance in prescribing OM and not introducing non-essential therapy during the trial is sought. Participants are also seen by MDs to confirm eligibility. All participants receive PMM, which is provided by MDs in the fifth week. Participants then receive EA, sham EA or no-EA for 10 weeks. Those who are allocated to the no-EA PMM alone group are given the opportunity to have EA treatment at the end of the 10-week treatment period. Participants in the EA and sham EA groups are followed-up for three months.

### Electroacupuncture interventions

The selection of acupuncture points is semi-structured. Unilateral Shousanli LI10 and Hegu LI4 (on the forearm and hand), and Zusanli ST36 and Fenglong ST40 (on the leg) will be used for EA. Up to eight supplementary acupuncture points will be chosen according to the adverse effects of OM that participants experience during that week. These points are manually needled. In total, up to 12 needles are used for each session. Disposable acupuncture needles of 0.25 mm diameter and 30 or 40 mm length (Hwato, Suzhou Medical Instrument Factory, China) are used.

The real and sham acupuncture procedures follow those described in our previous study [19] and the Standards for Reporting Interventions in Controlled Trials of Acupuncture [24]. Briefly, in the EA group, after needle insertion and de qi sensation is produced, described as a numb, distended and aching sensation, a battery operated electroacupuncture instrument (Model E600 HAN Multi-Purpose Digital Electronic Acupunctoscope, manufactured by Tens Plus Industrial Company, Hong Kong) is connected to the handles of four needles in the main acupuncture points in the extremities to deliver electrical stimulation for 20 minutes. Electrical stimuli will be delivered at an alternating frequency of 2 and 100 Hz every 3 seconds. The intensity of stimulation is strong but comfortable, and adjusted once during the treatment.

For sham EA, a set of sham points are developed to match each real point. Needles are shallowly inserted into the sham points without manipulation. A manufacture modified non-functioning EA stimulator showing a continuously flashing light and emitting a beeping sound is connected to the end of the four needles via wires, and placed on a table within the participant’s sight. De qi sensation is not intended. We have used this procedure previously, and participants could not tell EA from sham EA [19].

The treatment will be given twice a week for four weeks followed by once a week for two weeks then once every two weeks for four weeks. In total, 12 sessions should be delivered within 10 weeks. Participants completing less than 50% of the treatment are considered as non-compliant. Their data will be treated as missing data.

### Pain medication management

PMM is an integral part of pain management in multidisciplinary clinics. In this trial, PMM consists of patient education about pain and pain medications, and advice on OM reduction. A *Pain and Medication Management Information Brochure* has been developed to standardise PMM across all centres. In the fifth week, MDs explains to participants the impact of chronic pain, and the potential problems associated with OMs. OM reduction schedules are developed and explained to each participant at the interview. The schedule is flexible and individually tailored. It includes instructions for which type of OM to reduce first, how much and when, the alternative medications and what to do if pain increases. In general, short-acting OMs are reduced first, then the long-acting (slow-release) OMs. Participants are asked to reduce their OM dosage by 30% in week 8, 50% by week 11 and 75% to 100% by Week 14. Participants are asked to reduce OM as long as their pain is not worse. If increased pain causes distress or dysfunction, participants can increase their OM dose to the previous level or take alternative non-opioid medications prescribed by MDs. Clonidine may be prescribed for participants experiencing opioid withdrawal symptoms as judged by the MDs. The dosages of all pain relief medications are recorded.

As part of PMM, during the 10-week treatment and the three-month follow-up periods, a trained researcher will make a phone call to all participants three times during the treatment and once a month during the follow-up period to remind participants of the OM reduction schedule. During the trial period, participants are instructed to continue their routine therapies.

### Therapists

EA is provided by registered acupuncturists with at least three years of clinical experience. PMM is given by MDs at the pain management centres. All therapists undergo pre-trial training regarding the trial procedures. An *Acupuncture Treatment Manual* has been developed for training acupuncturists; and the *Pain and Medication Management Information Brochure* is used for training MDs.

### Co-interventions

Co-interventions are discouraged. Participants using other therapies for CMP such as herbal medicine, physiotherapy, chiropractic and osteopathy are required to either discontinue them before entering into the trial or maintain their use during the trial and record use in their diaries.

### Withdrawal

Participants who find their pain significantly worse or cannot tolerate EA may terminate their involvement in the study. They are assessed by one of the MDs with advice on alternative management strategies for their condition. Their data will be treated as missing data.

### Assessment of efficacy

A list of outcome measurement tools and when they are administered are listed in Table 1.

**Table 1:** Outcome measures to be administered throughout the trial

**Outcome measures**	**Components measured**	**Baseline**	**Treatment period**			**Follow-up period**		
		**W1**	**W5**	**W10**	**W14**	**W18**	**W22**	**W26**
**Questionnaires to assess outcomes of the treatment**								
Diary	Pain OM use non-OM use	✓ W1 to W4	✓	✓	✓	✓	✓	✓
Beck Depression Inventory	Mental status	✓	✓	✓	✓	✓	✓	✓
SF-36v2	Quality of life		✓	✓	✓	✓	✓	✓
Roland Morris Disability Questionnaire	Functionality		✓	✓	✓	✓	✓	✓
Short Opiate Withdrawal Scale	Withdrawal symptoms		✓	✓	✓	✓	✓	✓
Survey of patients’ attitude	Changes of attitudes		✓	✓	✓	✓	✓	✓
Chinese Medicine Chronic Pain Questionnaire	Pain and non-pain signs and symptoms	✓	✓	✓	✓			
**Questionnaires to assess credibility, expectancy and attitude to acupuncture or pain medications**								
Perception of EA treatment			✓(W6)		✓			
Acupuncture Expectancy Scale			✓(W5)					
Knowledge of Acupuncture		✓						
Pain Medication Questionnaire		✓						
**Elements of neuropathic pain**								
S-LANSS			✓(W5)					

OM: opioid medication; EA: electroacupuncture; SF-36v2: 36-item Short-Form Health Survey version 2; S-LANSS: self-report Leeds Assessment of Neuropathic Symptoms and Signs; W: week.

#### *Primary outcome measure*

The primary outcome measure is the change in OM between baseline and the end of the treatment period. OM usage is recorded daily in the Medication and Pain Diaries during the run-in period (between the first and fourth weeks), treatment period (between the 5^th^ and 14^th^ weeks) and follow-up period in the 18^th^, 22^nd^ and 26^th^ weeks. To make meaningful comparisons on medication use, the dosage of OM will be converted into morphine equivalent dose for data analysis [25].

#### *Secondary outcome measures*

Secondary outcome measures assess pain; impact of pain including function, quality of life and depression; consumption of non-opioid medications; and participants’ attitude to pain. All instruments have been widely used and demonstrated reliability and validity in the chronic pain population.

The outcome measures are as follows:

1. Number of participants who achieve 50% OM reduction at the end of the treatment and follow-up periods.

2. Type, severity and incidence of OM-related common AEs. Common AEs are somnolence, mental clouding, nausea and constipation. Uncommon AEs are fatigue, itching, adverse mood changes and dry mouth. They are assessed using visual analogues scale (0 = no symptom at all, 10 = very severe) to indicate the severity of these events.

3. Consumption of non-opioid medications.

4. Pain intensity measured with visual analogue scale (0 = no pain; 10 = worst pain possible) in the Medication and Pain Diary.

5. Quality of life assessed with 36-item Short-Form Health Survey version 2 (SF-36) [26].

6. Severity of depression assessed with the Beck Depression Inventory [27].

7. The level of function assessed with the Roland Morris Disabilities Questionnaire [28].

8. The presentation and severity of withdrawal symptoms assessed with Short Opiate Withdrawal Scale [29].

9. Participants’ attitude of pain assessed with Survey of Patients’ Attitude [30].

10. Chinese Medicine Chronic Pain Questionnaire, which we have developed to assess pain and non-pain signs and symptoms.

To make meaningful comparisons of non-opioid medications usage, the dosage of such medications is calculated according to Medication Quantification Scale Version III [31]. The Perception of EA Treatment Questionnaire [18] is given to the two acupuncture groups to assess the success of blinding in the 6^th^ and 14^th^ weeks. We also use Self-reported Leeds Assessment of Neuropathic Symptoms and Signs [32] to assess the certainty of neuropathic component of the pain.

### Assessment of safety and report of serious adverse events

An AE record form is attached to the end of the Medication and Pain Diaries so that participants can record any unexpected signs, symptoms and feelings during and after the treatment period. Acupuncture-related AEs are dizziness, fatigue, pain, bruising and infection. These will be scored using six-point scales (0 = none, 1 = minimal, 2 = mild, 3 = moderate, 4 = severe, and 5 = extremely severe). Participants are educated to differentiate AEs that are acupuncture-related from those that are OM-related. The former is usually short-lasting, whereas the latter persists when OM is consumed. Similarly, the acupuncturists will be required to record any adverse reactions during and after each treatment session. The likelihood that serious AEs reported by participants and acupuncturists are EA-related or OM-related will be assessed by two team members (ZZ, RH). They also consult, as appropriate, with the participant, MDs, and/or acupuncturists. The result of the assessment is recorded.

### Data storage and access

All participants’ files are kept in a coded order in secure locked cabinets. Consent forms and personal information are stored separately from study questionnaires. Data in electronic files are de-identified and files are stored as password protected intranet files at the trial administration centre. Only authorized research personnel have access to hard and soft copies of the files. The information is retained for 15 years. After that information will be shredded and disposed of according to university procedure. Only aggregated data will be presented in publications.

### Statistical analysis of the trial outcomes

The Statistical Program for Social Science (SPSS, the latest version) is used for data analyses. Baseline categorical data, demographic characteristics such as gender and age category, and numerical data are analysed with *χ*^2^ tests and one-way analysis of variance, respectively, to determine comparability of the three groups on these variables.

Linear mixed models (for continuous outcome variables) and generalised estimating equations (for categorical outcome variables) are used to examine the changes over time in weekly dosage of OM, numbers and types of OM-related AEs, severity of pain and Beck Depression Inventory. *χ*^2^ tests are also used to detect group differences in the numbers of dropouts and AEs.

Both intention-to-treat and per protocol analyses are used. Data from participants who do not violate the treatment protocols will be included in the per protocol analysis. Missing data are dealt with using the last value carried forward method. Missing Beck Depression Inventory and SF-36 data are dealt with according to the relevant manual.

Apart from the effects of types of intervention, the influence on the outcomes of differences between centres and therapists is also analysed. The relationship between OM reduction and attitude to pain are explored.

## Discussion

CMP is prevalent, with high socioeconomic impacts. OMs have been increasingly prescribed for patients with CMP both in Australia and worldwide. There is, however, no evidence supporting the long-term use of opioids for patients with CMP. Furthermore, OM is associated with severe AEs, which further impact on patients’ quality of life. OM might be a hindrance that prevents patients from taking part in more active management programmes. Effective treatments that reduce OM consumption and related AEs are urgently needed and will benefit both patients and society.

Our pilot study shows that EA, when combined with PMM, is potentially effective in reducing OM usage by patients with chronic non-cancer pain. It also demonstrates the feasibility of a collaborative research involving complementary health professionals, clinicians in pain management, pain researchers and acupuncture researchers. Following the validated protocol, with an expanded team of experts and researchers, the proposed study will for the first time determine whether EA is of significant clinical value in assisting the management of debilitating chronic pain by reducing OM consumption and its associated AEs, as well as improving the quality of life of those with CMP.

Such an outcome will provide a rationale for incorporating EA into existing multidisciplinary pain management programmes. Future research will examine whether a different form of EA, such as non-invasive EA, can be used by patients at home to achieve the same effectiveness.

## Trial status

The trial is open to recruitment and will be completed by the end of December 2012. We expect the results will be published by the end of 2013 or early 2014.

## Abbreviations

AEs: adverse events; CMP: chronic musculoskeletal pain; EA: electroacupuncture; MD: medical doctor; OM: opioid medication; PMM: pain medication management; SF-36: 36-item Short-Form Health Survey version 2.

## Competing interests

The authors declare that they have no competing interests.

## Authors’ contributions

RH and ZZ initiated and developed the study, and contributed to the design of the study and the protocol. CCL, SG, MH, CA, AAS, CDC, YYW and SCL contributed to the development of the trial protocol. All authors have read and approved the final manuscript.
